# Evaluating the impact of engaging healthcare providers in an AI-based gamified mHealth intervention for improving maternal health outcomes among disadvantaged pregnant women in Lebanon

**DOI:** 10.3389/fdgth.2025.1574946

**Published:** 2025-08-12

**Authors:** Shadi Saleh, Nour El Arnaout, Nadine Sabra, Asmaa El Dakdouki, Khaled El Iskandarani, Zahraa Chamseddine, Randa Hamadeh, Abed Shanaa, Mohamad Alameddine

**Affiliations:** ^1^ Global Health Institute, American University of Beirut, Beirut, Lebanon; ^2^ Faculty of Health Sciences, American University of Beirut (AUB), Beirut, Lebanon; ^3^ Primary Healthcare and Social Health Department, Ministry of Public Health (MOPH), Beirut, Lebanon; ^4^ United Nations Relief and Works Agency for Palestine Refugees in the Near East (UNRWA), Beirut, Lebanon; ^5^ College of Health Sciences, University of Sharjah, Sharjah, United Arab Emirates

**Keywords:** maternal health, gamification, artificial intelligence, mHealth, neonatal outcomes, antenatal care (ANC), healthcare provider

## Abstract

**Introduction:**

Maternal health in Lebanon faces significant challenges, particularly among disadvantaged populations, due to limited access to antenatal care (ANC) and a strained healthcare system. While mHealth interventions have improved maternal outcomes globally, few engage healthcare providers (HCPs) or incorporate advanced tools like artificial intelligence (AI) and gamification. This study evaluated the effectiveness of an AI-based, gamified mHealth intervention, Gamification and AI and mHealth Network for Maternal Health Improvement (GAIN MHI), on ANC utilization and maternal and neonatal outcomes in Lebanon.

**Methods and materials:**

The intervention included two arms: one targeting pregnant women and their spouses without HCP engagement and another involving HCPs. A post-intervention analysis was conducted with 2,880 pregnant women divided into three groups: control (*n* = 1,315), non-HCP intervention (*n* = 668), and HCP intervention (*n* = 897). Intervention components included AI-driven, gamified HCP professional development via the GAIN MHI app, weekly WhatsApp-based educational messages, and ANC visit reminders. Data on healthcare access (ANC visits, supplement intake, ultrasounds, and lab tests) and outcomes (term delivery, maternal/neonatal complications) were analyzed using logistic regression to calculate adjusted odds ratios (OR).

**Results:**

The HCP arm significantly improved healthcare access, with higher odds of attending ≥4 ANC visits (OR = 1.968, 95% CI: 1.575–2.459), completing ≥2 ultrasounds (OR = 3.026, 95% CI: 2.301–3.981), lab test completion (OR = 2.828, 95% CI: 1.894–4.221), and supplement intake (OR = 1.467, 95% CI: 1.221–1.762). Term deliveries were more likely in the HCP arm (OR = 1.360, 95% CI: 1.011–1.289), and neonatal morbidity decreased by 52.15% (OR = 1.521, 95% CI: 1.127–2.051). No improvements were seen in abortion rates, and normal deliveries decreased across intervention arms. Significant baseline demographic differences, including nationality and chronic disease prevalence, were observed between groups.

**Discussion:**

Integrating HCPs into an mHealth intervention significantly enhanced ANC uptake and maternal and neonatal outcomes in disadvantaged populations in Lebanon. These findings underscore the importance of combining digital tools with clinical support to address systemic barriers and improve maternal health in resource-limited settings. Future interventions should address delivery practices and broader social determinants of health to achieve sustainable impacts.

## 1 Introduction

Maternal health is a critical public health issue affecting the lives of many mothers across the globe, especially in Low- and Middle-Income Countries (LMICs). Significant efforts have led to reducing maternal mortality by 34% from 2000 till 2020 (1–4). However, 287,000 women died globally in 2020, with 95% of deaths occurring in LMICs (4). Lebanon, as an LMIC, reflects this global disparity, especially within its disadvantaged populations, including Syrian and Palestinian refugees (5–7). Pregnant women in Lebanon face a high maternal mortality rate (MMRs) and a range of pregnancy-related complications, including anemia, hemorrhage, urinary tract infections, and gestational diabetes (5–7). These complications are closely linked to limited access to essential maternal services including antenatal care (ANC), which is vital for preventing and managing complications during pregnancy (7–9). A variety of socio-economic and healthcare system barriers significantly hinder women's access to ANC in Lebanon (7, 10, 11). Among these obstacles is the lack of social and spousal support, which often discourages pregnant women from seeking care (7, 12–14). Financial constraints further limit their ability to afford ANC services (7). These challenges are exacerbated by the country's healthcare system, which is severely strained due to an ongoing economic crisis, the influx of refugees, and shortages in skilled healthcare providers (HCPs) and medical supplies (5–7, 10, 15, 16). The interplay of these social, economic, and healthcare challenges underscores the urgent need for targeted, comprehensive interventions to address maternal health challenges in Lebanon (17, 18).

The widespread use of mobile phones in LMICs has paved the way for innovative, cost-effective, and scalable mobile health (mHealth) interventions (19–21). These interventions have become a key element of global strategies aimed at reducing maternal mortality and improving maternal health outcomes, especially in underserved communities (19, 22–24). mHealth solutions have shown effectiveness in addressing gaps in access to healthcare services for pregnant women (19, 23). Studies have demonstrated that mHealth interventions, such as SMS or mobile-based messaging, significantly enhance access to ANC by providing timely information and reminders (23, 25–28). For instance, the mMitra intervention, which delivers pregnancy-related voice messages, has yielded an improvement maternal healthcare practices and outcomes (29, 30). Similarly, mhealth interventions that engage spouses during pregnancy have been shown to promote positive health behaviors among expectant mothers (21, 31, 32). The Mobile Alliance for Maternal Action (MAMA) exemplifies such efforts by targeting both pregnant women and their spouses (32). This program delivers educational messages on maternal, newborn, and child health, enhancing healthcare-seeking for women and their infants up to one year old (33). Further research highlight the potential of mHealth solutions to improve maternal health and expand access to essential services (26, 34, 35). While these interventions have shown promise, maternal health and healthcare-seeking behaviors are influenced by a combination of health, social, and environmental factors (17, 36). Therefore, efforts to enhance these areas must go beyond family dynamics and adopt more inclusive and collaborative approaches that engage broader support networks, such as HCPs, among others (17, 36).

In contexts where healthcare-seeking behaviors are deeply influenced by HCPs attitudes, there is a pressing need for interventions that engage women alongside their HCPs to improve maternal health outcomes (17, 36). Studies have shown that tailored interventions engaging HCPs improve women's compliance with care (37, 38). These HCPs play a vital role in delivering quality care, education, and guidance to women and their families, and addressing key concerns during pregnancy and the postnatal phase (17, 39, 40). Their knowledge and communication skills are crucial for building trust, enhancing access to services, and fostering a supportive environment for expectant mothers (17, 36). Continuous learning for HCPs is therefore essential to maintaining and improving the quality of health care, including maternal health (41).

mHealth interventions targeting HCPs for maternal health in LMICs have primarily focused on clinical decision support and enhancing communication both among HCPs, and between HCPs and patients, with limited emphasis on improving HCP knowledge and skills (26, 42–46). For instance, programs like Wired mothers in Tanzania and maternal and child health application (MatHealthApp) in Uganda have successfully enabled two-way communication between pregnant women and HCPs (43, 47). Recent advancements in mhealth have shifted towards using gamification, allowing HCPs to participate in interactive learning and address clinical challenges (46, 48). A notable application is the Safe Delivery App (SDA), which is designed to enhance HCPs skills, with an upgraded version that incorporates gamification to motivate users and create an interactive learning experience (49, 50). Similarly, the Maternal and Newborn Technology for Resilience (MANTRA) app employs gamified elements to boost awareness of risk factors related to maternal and child health (51). Users found the gamified approach engaging, leading to greater understanding of maternal and child health issues and more informed choices (51).

Aiming to improve care quality and tackle a variety of health challenges, the integration of artificial intelligence (AI) into the healthcare sector has been on the rise (52, 53). AI applications have been used to assist in diagnostics, extend virtual healthcare services to underserved areas, and fill gaps in knowledge and skills (54). In the context of maternal health, AI has demonstrated considerable potential by forecasting risks such as pre-eclampsia, postpartum depression, gestational diabetes, anemia, stillbirths, preliminary signs of miscarriage, and preterm births (55, 56). Additionally, AI increases access to maternal health services and enables HCPs to make informed decisions regarding the pregnancy process, including determining when to perform a C-section or induce labor (57–59). AI technology has also demonstrated potential in enhancing the education and learning of HCPs by accelerating the training of HCPs in evaluating preterm births (60).

mHealth interventions are increasingly being employed to address the diverse health needs of vulnerable populations in Lebanon. These technologies have been instrumental in managing non-communicable diseases (NCDs) and enhancing primary healthcare services for both vulnerable Lebanese communities and refugee populations. Through tools such as educational text messages, appointment reminders, and HCP capacity-building guidelines, mHealth has improved the delivery of healthcare in these settings (61–64). One notable mHealth innovation is the “Distance Support Program” (DSP), which has been used to improve palliative care in Lebanon. DSP adopts a collaborative approach to support caregivers of patients who are geographically distant by utilizing phone calls, video calls, and messaging to ensure continuity of care (65).

Regarding maternal health, Lebanon's efforts remain limited in implementing mHealth solutions that significantly improve maternal health outcomes and increase ANC visits (66). One impactful intervention focuses on maternal health for Palestinian refugees, including those residing in Lebanon. The United Nations Relief and Works Agency (UNRWA)-developed electronic mother and child health (e-MCH) application that integrates a detailed database on maternal and infant care with appointment reminders, ensuring that essential healthcare services remain uninterrupted (67). Existing mHealth interventions, though beneficial, primarily focus on individual care delivery and lack integration between pregnant women and their HCPs. The absence of solutions that actively engage both pregnant women and HCPs represents a critical gap in Lebanon's maternal health interventions. The country has yet to fully explore AI-powered solutions or gamification techniques, which represents a significant gap in the use of advanced technologies to enhance maternal health outcomes (68). Bridging this gap requires comprehensive, collaborative mHealth interventions that engage not only mothers and their families but also their HCPs, ensuring a more holistic and effective approach to maternal health.

This study aims to assess the impact of including HCPs in an AI-based gamified mHealth intervention, titled ‘Gamification and Artificial Intelligence and mHealth Network for Maternal Health Improvement’ (GAIN MHI), on the maternal health outcomes of disadvantaged pregnant women in Lebanon.

## 2 Methods and materials

### 2.1 Study design

This study is a community interventional trial with historical control conducted at different primary healthcare centers (PHCs). Historical controls were not recruited concurrently in this study as the treatment group but rather from existing medical records at each center which were randomly assigned to either the intervention or control centers. The study was implemented in three phases: the pre-intervention phase (July 2021–December 2021), used to establish baseline data not included in the analysis, the intervention phase, which occurred from September 2021 to December 2022, and the post-intervention phase, which took place from March 2023 to June 2023. Data was collected by research assistants at each center and included demographics, medical and obstetric histories, as well as maternal and neonatal outcomes and complications.

### 2.2 Centers selection

This study took place at twenty PHCs across five governorates in Lebanon—Beirut, Bekaa, North, South, and Mount Lebanon. One PHC was excluded due to logistical constraints. Primary Health Centers (PHCs) were allocated equally between intervention and control arms, with ten sites per arm. Within each arm, half of the PHCs (five) were UNRWA-operated centers serving exclusively Palestinian refugees, and the other half (five) were non-UNRWA facilities from the Lebanese Ministry of Public Health network serving disadvantaged Lebanese and non-Lebanese populations (e.g., Syrian). To control for geographic variability, all 20 PHCs were first matched into ten regional pairs, five pairs of UNRWA centers and five pairs of non-UNRWA centers, where each pair was located within the same region. Within each matched pair, one PHC was randomly assigned to the intervention arm and the other to the control arm, ensuring that both study arms included an equal mix of UNRWA and MOPH-governed facilities. Of the remaining centers, ten served as controls and nine as interventions.

### 2.3 Study population

The target population included pregnant women of all ages who visited the selected PHCs during their first trimester within the intervention period and continued to attend the selected PHCs for at least one year. Women that met this inclusion criteria were involved in the study. The post-intervention phase comprised pregnant women who were already enrolled in the intervention and attending the PHCs throughout the intervention period, as well as those who visited the control PHCs within the same timeframe.

### 2.4 GAIN MHI intervention

#### 2.4.1 About the intervention

**Figure 1:**
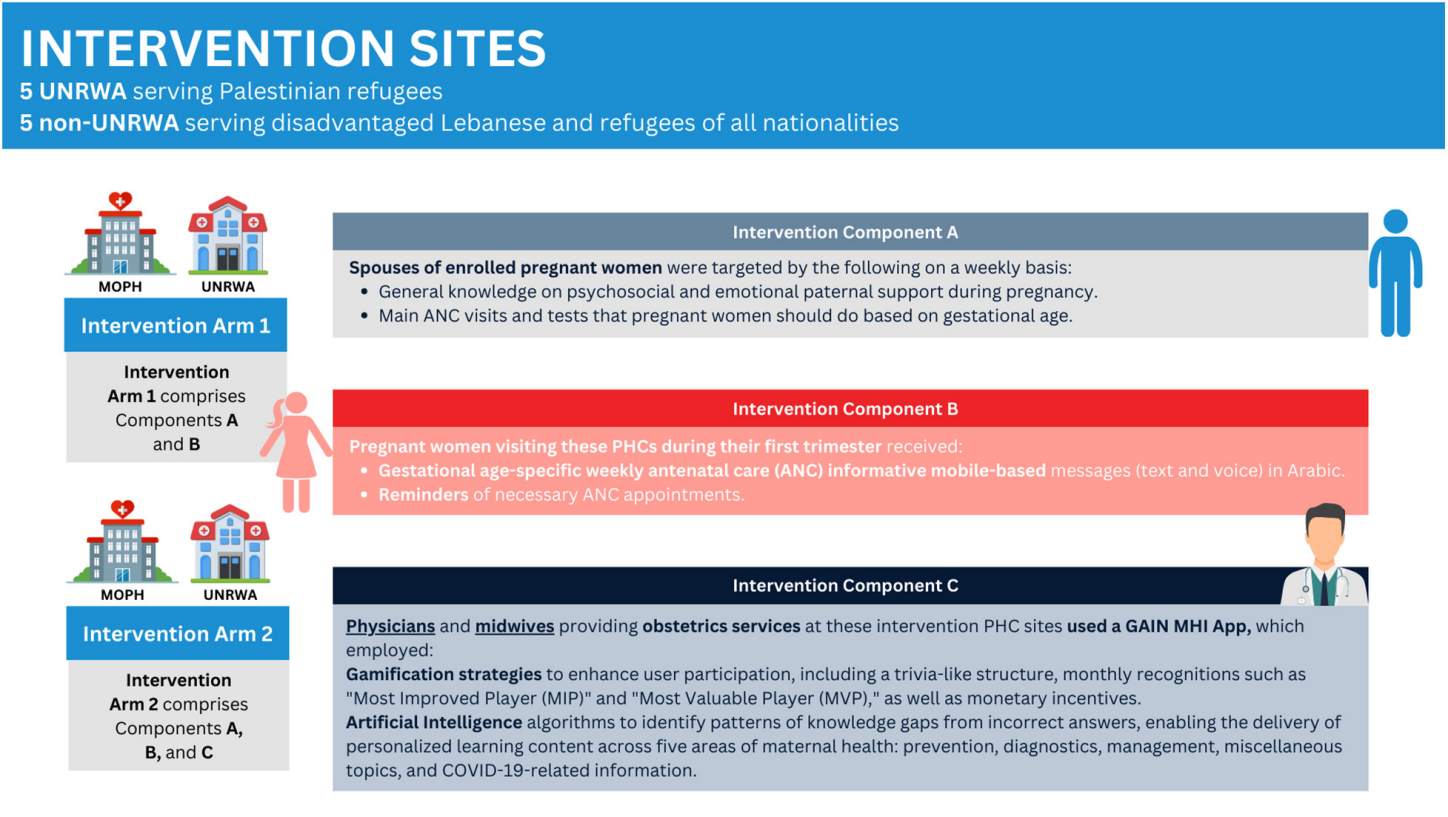
Visual representation of the different components in the intervention arms. Created using Canva. Illustrations from Canva, licensed under Pro Content License.

The GAIN MHI intervention (Figure 1) was divided into two arms: one included HCPs alongside pregnant women and their spouses, while the other involved only pregnant women and their spouses. Two key components distinguish this intervention: mobile-based messages (text and voice) and a mobile application (GAIN MHI App), both designed to improve maternal health outcomes. The intervention was standardized across all PHCs, with no site-specific adaptations implemented at any point.

The mobile-based messages targeted pregnant women in their first trimester, particularly Syrian and Palestinian refugee women, as well as Lebanese women attending PHCs serving disadvantaged populations. These were weekly gestational-age specific messages delivered through WhatsApp in Arabic, both as text messages and voice messages. The messages provided educational content tailored to gestational age, covering pregnancy education, nutritional guidance and supplementation, danger signs, and postpartum care for up to six months. Additionally, they promoted postpartum visits, breastfeeding, contraception, maternal mental health, and infant vaccinations. Reminders for ANC appointments, lab tests, and medical assessments were also sent. Our intervention targeted the spouses of the enrolled pregnant women as well. The spouses received weekly messages that are tailored to the gestational age of the wife, emphasizing the importance of psychosocial and emotional support during pregnancy.

In parallel, the GAIN MHI App was designed and developed by the Global Health Institute (GHI) at the American University of Beirut (AUB). The GAIN MHI App targeted HCPs by integrating gamification and AI concepts to support their professional development. To boost user participation, the App incorporated game-like elements, such as trivia-like structure, monthly recognition (most improved player (MIP), most valuable player (MVP), and monetary incentives. AI algorithms within the App identified knowledge gaps based on incorrect answers, allowing for personalized learning in five areas of maternal health: prevention, diagnostic, management, miscellaneous, and COVID-19-related questions. Questions were randomly selected through a spin wheel, and users were allowed to spin the wheel 30 times per month. Users were required to select an answer, and a corresponding explanation would show up upon submission. Additionally, the app had a knowledge resource center (KRC) and sent to users automated WhatsApp messages with feedback on scores, answers, remaining attempts, monthly performance, and rewards. The App was made available in both English and Arabic languages and represented a new way to improve healthcare education and maternal healthcare.

### 2.5 Data collection

Medical records of the study participants were reviewed to collect data during the post-intervention phase, which occurred from March 2023 to June 2023. For women, who experienced multiple pregnancies during the study period, only the record of their latest pregnancy, if it met the study's inclusion criteria mentioned previously, was collected. Data collected included the following: demographics, medical and surgical history, pregnancy history (supplements, ANC vaccinations, the first lab tests and ultrasounds images done, and maternal complications if any), neonatal complications, and breastfeeding history.

### 2.6 Ethical approval

Ethical approval was sought and obtained from the Institutional Review Board at the American University of Beirut (Protocol number: SBS-2020-0317) and the respective ethical committees at MOPH and UNRWA.

### 2.7 Study parameters

Study parameters were extracted from the medical records for all pregnant women at both data collection phases (pre and post intervention) in all the centers. Table 1 shows the set of indicators that were selected to monitor the effectiveness of the intervention along with their definitions. The selected parameters were categorized into two domains: access to healthcare, and maternal and neonatal outcomes. Access to healthcare included completing more than 4 ANC visits, completing at least 2 ultrasound images (both in accordance with WHO recommendations) (69), completing the available laboratory tests at the PHCs and taking supplements during pregnancy. Having onset of delivery on term, having a normal delivery type, reduction in abortion, reduction in any neonatal morbidity and any maternal complication were the indicators comprising the domain of maternal and neonatal outcomes.

**Table 1:** Selected indicators to assess the effectiveness of the intervention on access to care, maternal and neonatal outcomes.

Domain 1: access to healthcare	Definition
Antenatal care visits	Percentage of pregnant women who completed 4 or more antenatal care visits (ANC) based on the WHO recommendation (8)
Supplements intake	Percentage of pregnant women taking the all the required vitamins that a women should take including Folic acid, Iron, and Calcium (82, 83)
Ultrasounds images	Percentage of pregnant women who completed 2 or more ultrasounds images through their pregnancy period (84)
Lab test	Percentage of pregnant women who completed both the urine analysis and the available blood tests at their PHCs including Fasting Blood Sugar (FBS), blood group ± Rh, and CBC (85, 86)
Domain 2: maternal and neonatal outcomes	
Onset of delivery	Percentage of pregnant women who had term delivery; delivering their latest child after the week 37 (87)
Method of delivery	Percentage of pregnant women who had normal delivery
Abortion	Percentage of women who had abortion in their latest pregnancy before the week 22 (88)
Neonatal morbidities	Percentage of pregnant women who faced any neonatal complication including either neonatal intensive care admission (NICU admission), preterm delivery, iatrogenic preterm birth injury, low birthweight, and neonatal early death
Maternal complications	Percentage of pregnant women who had faced any maternal complication including either: gestational hypertension, gestational diabetes, perinatal depression, preterm labor, stillbirth, abortion, and post-partum hemorrhage

### 2.8 Statistical analysis

Pregnant women being Lebanese, Syrian, or Palestinian who participated in the post-intervention phase were included in the analysis. Demographic and health characteristics were summarized for the two intervention arms (HCP and non-HCP) and the control group using counts and percentages or means and standard deviations. Chi-square tests and independent sample t-tests were employed to assess the existence of possible characteristic differences across the control and the intervention arms. Bivariate logistic regression was generated to calculate the unadjusted odds ratios aimed to quantify the proportion discrepancies in study indicators between each intervention arm and the control group. Separate multivariate logistic regression models were developed for every indicator, comparing the intervention arms in reference to the control group while controlling for potential differential characteristics that appeared to be significant. Analysis was carried out at 0.05 significance level using the Stata SE software program.

## 3 Results

A total of 2,880 pregnant women participated in the post-intervention phase, divided as follows: 1,315 in the control group, 668 in the non-HCP intervention arm focusing on pregnant women and their spouses (with no involvement of healthcare professionals), and 897 in the HCP intervention arm, which included both pregnant women and, their spouses whenever possible, with the intervention provided by healthcare professionals. The selected study indicators which were used to assess the effectiveness of the intervention are depicted in Table 1. Table 2 shows the **demographics and health characteristics** of the pregnant women across the **control** and the **two interventional arms**. The age distribution varied differentially across the control group and intervention arms, with the highest percentage of women aged between 18 and 29 years in all of them (*p* = 0.009*). **Nationality distribution** varied significantly as well, Palestinian participants were primarily in the control group (27.0%), while the majority of both intervention arms non-HCP and HCP consisted of Syrian refugees (78.14% and 64.66% respectively), highlighting a significant difference across the control group and intervention arms (*p* < 0.05*). In terms of **health characteristics,** the control group exhibited a higher prevalence of chronic diseases (33.46%) compared to the non-HCP (10.08%) and HCP (31.82%) arms (*p* < 0.05*). The HCP intervention arm had a higher proportion of participants with a **history of previous pre-term births** (5.02%) compared to the control group (2.97%) and non-HCP arm (1.05%) (*p* < 0.05*). Additionally, the HCP intervention arm had a higher rate of previous **pre-term births** (5.02%) (*p* < 0.05*) and higher number of previous deliveries (gravida) of 2.78 on average, slightly lower than the control group (2.97) (***p*** **=** 0.037*). Overall, these findings highlight notable differences in age, nationality, health conditions, and reproductive histories between the control group and the intervention arms.

**Table 2:** Demographic and health characteristics of study participants by study groups (*n* = 2,880).

	Control *n* = 1,315 *n* (%)		Non-HCP intervention arm *n* = 668 *n* (%)		HCP intervention arm *n* = 897 *n* (%)		*p*-value
Age							
<18	42	(3.19)	8	(1.20)	16	(1.78)	0.009^a^
18–29	696	(52.93)	348	(52.10)	474	(52.84)	
30–39	508	(38.63)	278	(41.62)	379	(42.25)	
>40	69	(5.25)	34	(5.09)	28	(3.12)	
Nationality							
Lebanese	242	(18.40)	81	(12.13)	108	(12.04)	<0.05^a^
Palestinian	355	(27.00)	65	(9.73)	209	(23.30)	
Syrian	718	(54.60)	522	(78.14)	580	(64.66)	
Chronic Diseases, yes	439	(33.46)	67	(10.08)	281	(31.82)	<0.05^a^
Previous Pre-term, yes	39	(2.97)	7	(1.05)	45	(5.02)	<0.05^a^
Previous abortion, yes	340	(25.86)	179	(26.80)	257	(28.65)	0.345
Previous C-Section, yes	373	(34.51)	195	(29.19)	252	(28.70)	0.010^a^
Mean Gravida (SD)	2.97	(1.93)	2.82	(1.76)	2.78	(1.73)	0.037^a^

^a^ Significant at 0.05.

### 3.1 Access to healthcare

Table 3 presents the bivariate and multivariate analyses for each study indicator, showcasing both unadjusted and adjusted odds ratios. The differences in proportions of the selected indicators across the control group and the two intervention arms are quantified through the unadjusted odds ratios. Separate logistic regression models were employed for each indicator to compute adjusted odds ratios (OR) for the intervention arms compared to the control group, while controlling for potential confounding factors including age, nationality, history of chronic diseases, pre-term delivery, cesarean section, and gravida. Results indicated that the HCP intervention arm experienced a 96.8% increase in the odds of attending four or more ANC visits compared to the control group (adjusted OR = 1.968, 95% CI: 1.133, 1.832), which is greater than the 44.1% increase observed in the non-HCP intervention arm (adjusted OR = 1.441, 95% CI: 1.133, 1.832). A significant improvement was observed only in the HCP intervention arm, where pregnant women were 3.02 times more likely to complete two or more ultrasound images compared to the control group (adjusted OR = 3.026, 95% CI: 2.301, 3.981). In regard to **lab tests,** both intervention arms demonstrated significantly improved odds of completing the required lab tests compared to the control, with the HCP intervention arm showing greater odds (adjusted OR = 2.828, 95% CI: 1.879, 4.190) than the non-HCP intervention armPs (adjusted OR = 2.537, 95% CI: 1.653, 3.832). For the supplement intake, the odds of taking the required supplements were significantly higher in the HCP intervention arm, with an adjusted OR of 1.467 (95% CI: 1.221, 1.762).

**Table 3:** Proportion (%), unadjusted and adjusted odds ratio (OR), 95% confident interval (CI), for study indicators across intervention arms in reference to the control group.

	Control *n* = 1,315		Non-HCP intervention arm *n* = 668			HCP intervention arm *n* = 897		
	%	OR	%	OR^a^	OR^b^ (95%CI)	%	OR^a^	OR^b^ (95%CI)
Access to healthcare								
ANC visits								
<4 visits	25.48	1	19.06	-	-	22.53	-	-
≥4 visits	74.52	1	80.94	1.254^c^	1.441^c^(1.133, 1.832)	77.47	1.451^c^	1.968^c^(1.575, 2.459)
US images								
<2 images	19.53	1	19.13	-	-	10.18	-	-
≥2 images	80.65	1	80.87	1.014	1.201 (0.925, 1.559)	89.82	2.116^c^	3.026^c^(2.301, 3.981)
Completed lab tests								
No	38.25	1	23.05	-	-	27.42	-	-
Yes	61.75	1	76.95	2.067^c^	2.537^c^(1.664, 3.856)	72.58	1.639^c^	2.828^c^(1.894, 4.221)
Took the required supplements								
No	35.36	1	16.92	-	-	30.14	-	-
Yes	64.64	1	83.08	0.521^c^	0.825 (0.669, 1.017)	69.86	0.945	1.467^c^(1.221, 1.762)
Maternal and neonatal outcomes								
Onset of delivery								
Pre-term	12.39	1	14.74	-	-	10.21	-	-
Term	87.61	1	85.26	0.818^c^	0.917 (0.673, 1.248)	89.71	1.232	1.360^c^(1.011, 1.829)
Method of delivery								
C-section	46.02	1	54.52	-	-	53.49	-	-
Normal	53.98	1	45.48	0.711^c^	0.694^c^(0.527, 0.914)	46.51	0.741	0.688^c^(0.539, 0.877)
Neonatal morbidities								
Yes	96.96	1	95.36	-	97-	93.98	-	-
No	3.04	1	4.64	1.066	1.220 (0.881, 1.688)	6.02	1.275^c^	1.521^c^(1.127, 2.051)
Abortion								
Yes	88.90	1	89.52	-	-	91.08	-	-
No	11.10	1	10.48	0.644	0.688 (0.416, 1.139)	8.92	0.489	0.554^c^(0.363, 0.848)
Maternal complications								
Yes	76.12	1	74.55	-	-	73.58	-	-
No	23.88	1	25.45	0.918	0.989 (0.783, 1.249)	26.42	0.873	0.994 (0.810, 1.221)

-Reference category.

^a^ Unadjusted OR.

^b^ Adjusted OR for potential differences in age, nationality, history of chronic diseases, pre-term delivery, c-section and gravida.

^c^ Significant at 0.05.

### 3.2 Maternal and neonatal outcomes

Only the HCP intervention arms demonstrated a significant improvement in **term deliveries** as compared to the control group after adjusting for confounders (adjusted OR = 1.360, 95% CI: 1.011,1.289). However, the odds of having **normal deliveries** were significantly lower in both intervention arms relative to the control with the adjusted OR for the non-HCP intervention arm at 0.694 (95% CI: 0.527, 0.914) and 0.688 (95% CI: 0.539, 0.877) for the intervention arm including HCPs. There was a 52.15% increase in the odds of not experiencing any **neonatal morbidity** after adjusting for confounders (OR = 1.521,95% CI: 1.127,2.051). Conversely, there were no improvements in abortion rates across the two intervention arms, with the likelihood of not having an abortion being lower in the HCP intervention arms (adjusted OR = 0.554, 95% CI: 0.363–0.848) compared to the control group.

## 4 Discussion

This study evaluated the impact of including HCPs into a mobile health and AI-based gamified intervention aimed at improving maternal health outcomes and increasing the uptake of ANC services among disadvantaged Lebanese and refugee pregnant women. The results across the two intervention arms were promising regarding the outcomes.

The findings demonstrated that the HCP intervention arm led to significant improvements in adherence to the required number of ANC visits, ultrasound and lab tests completion, supplement intake, and neonatal outcomes compared to the non-HCP intervention arm. However, neither intervention significantly influenced abortion rates, with the HCP arm showing a lower likelihood of avoiding abortion, possibly due to improved detection of medically necessary abortions (70).

### 4.1 Access to primary healthcare services

The improvement in ANC adherence observed in the HCP intervention arm aligns with prior studies highlighting the positive impact of mHealth interventions targeting the HCPs on improving ANC attendance and overall health (71, 72). This may be attributed to the added value of HCP involvement, which provides personalized, professional guidance and reassurance throughout the pregnancy journey. Abraham et al. discovered that the relationship between HCPs and patients in maternal care plays a crucial role in determining both the quality and amount of care women receive, with potential effects on maternal morbidity and mortality in low- and middle-income countries (36). These results suggest that integrating HCPs into mHealth interventions could further enhance their effectiveness in promoting ANC attendance, consistent with other research advocating for the combined role of digital health and human support in maternal health (36).

The proportion of women receiving two or more ultrasound images was comparable in both the control group and the non-HCP intervention arm. Conversely, the HCP intervention arm showed a significantly higher proportion of women completing two or more ultrasound images. This suggests that mhealth interventions alone may not significantly improve ultrasound attendance without direct healthcare provider involvement. While mHealth tools offer valuable information and reminders, their effectiveness in behavior change is limited without the engagement of HCP (36). This finding underscores the essential role of HCPs in prenatal care, as they encourage adherence to ultrasound protocols. Their involvement not only enhances the perceived importance of these procedures but also promotes compliance among pregnant women (36). Similarily, The likelihood of taking the required supplements was significantly higher in the intervention arm that included HCPs compared to the control group. Bangal et al. witnessed such increase in the uptake of supplements by pregnant women when HCPs were engaged in the mhealth intervention targeting maternal health (42). The increased compliance with laboratory testing among women in the intervention arm that included HCPs reflects the value of a comprehensive approach to maternal healthcare. By combining digital interventions with the expertise and support of HCP, we can create a more robust framework for improving maternal health outcomes. Mbunge & Sibiya found that engaging not only the women, but also their HCPs among other broader support networks is crucial to maximize the transformative potential of mhealth in maternal care (71). This integrated model will likely increase ANC visits, attendance rates for ultrasounds and lab tests, and compliance with supplements intake. It will also foster a deeper understanding of the importance of these services among pregnant women, ultimately contributing to healthier pregnancies and better long-term outcomes for both mothers and their infants.

### 4.2 Maternal and neonatal outcomes

The HCP intervention arm demonstrated significant improvements in term deliveries and a notable increase in the odds of avoiding neonatal morbidity. These outcomes may be attributed to several interconnected factors, particularly the targeted approach of the intervention, which enhanced HCPs theoretical knowledge through the GAIN MHI App. By integrating gamification and AI, the app enabled HCPs to continuously refine their expertise in maternal health, equipping them to manage complications more effectively and provide tailored support to pregnant women. Abraham et al. found that enhancing HCP knowledge in delivering care boosted women's trust and acceptance of care services, leading to improved care quality and better health outcomes (36). This increased knowledge likely translated into improved clinical practices and better adherence to antenatal care protocols.

The supportive relationship between HCPs and pregnant women is crucial in fostering adherence to health recommendations and enhancing overall pregnancy outcomes (73, 74). While the positive effects on neonatal morbidity and term deliveries are notable, the lack of significant improvement in maternal complications indicates the necessity for a more comprehensive approach. In addition to social determinants of health, such as socioeconomic status, education, and access to healthcare, other factors could contribute to maternal complications. These factors may include the lack of standardized protocols, variations in HCP training and experience, and potential gaps in continuity of care during the pregnancy journey, which make it harder to control maternal complications given that women face problems which are unpredictable (75). Even with enhanced HCP knowledge, pregnant women still face sudden, unpredictable complications that may lead to serious harm or death (1, 76). These, combined with systemic issues within the healthcare infrastructure, could have limited the ability to effectively manage maternal complications (1, 7, 77, 78).

The analysis also reveals that the likelihood of normal deliveries was significantly lower in both intervention arms compared to the control, which may be attributed to the rising rates of C-sections observed in the control group and intervention arms, a trend that is concerning on a global scale. The unnecessary increase in C-sections without clear medical indications can have negative health consequences and lead to inefficiencies in resource allocation (79–81). This suggests that while the interventions were effective in improving certain outcomes, they may not have fully addressed the complexity of delivery methods. Health system factors, cultural perceptions favoring C-sections, and possible selection bias may have influenced these results, limiting the interventions’ impact on delivery mode. The intervention, whether it included HCPs or not, did not yield significant improvements in abortion rates compared to the control group, indicating that mHealth interventions alone may not be sufficient to influence these outcomes. Financial constraints, in particular, may contribute to the decision to undergo abortion due to the substantial financial responsibilities associated with raising children. Women who have access to HCPs may be more likely to take an abortion decision and to carry it under medical supervision. These complexities highlight the need to consider broader systemic and cultural contexts when evaluating intervention effectiveness. This proposition begs for confirmation in future research.

In light of these findings, it is essential to adopt a multifaceted strategy that combines clinical excellence with efforts to mitigate social determinants of health. Addressing financial and social barriers alongside clinical care will be crucial for achieving more equitable improvements in maternal and neonatal health. Additionally, fostering standardized clinical protocols and ensuring continuity of care throughout the pregnancy journey will enhance the overall effectiveness of interventions.

### 4.3 Limitations

Several limitations were encountered in this study. As a chart review study, the quality and completeness of the data were dependent on the accuracy of medical records. Inconsistent or incomplete documentation of ANC details may have affected the reliability of the findings. In addition, retrospective data collection may have introduced bias if key health information is missing from the records. A concern that may be particularly pronounced in the context of Syrian refugees, where healthcare access is often fragmented, and medical records may be poorly maintained. Data entry errors could have occurred during the transfer of information from one source to another, leading to inaccuracies that may compromise the integrity of the dataset. To address this, field supervisors conducted regular supervision and random spot checks to ensure adherence to protocols and the accurate recording of participant responses. All data collectors received rigorous training prior to data collection to standardize procedures and minimize interviewer and recording bias.

## 5 Conclusion

In conclusion, the findings of this study underscore the critical role of involving HCPs in reinforcing the effectiveness of AI-based digital health interventions by providing personalized guidance and fostering trust. The coupling of HCP involvement with AI based gamified interventions showed improvement across various indicators related to improved access to care and better health outcomes. Since AI based gamified tools are relatively inexpensive and swiftly amenable to upscaling, it is recommended that such tools not only be utilized by all HCPs engaged with maternal health but also to invest in further developing such tools and in making them more comprehensive and user friendly. Practical scale-up requires infrastructure for digital access, standardized training for HCPs in digital health and AI, ongoing technical support to ensure effective integration into existing workflows, and sustainable financing mechanisms, such as public–private partnerships, donor programs, or insurance extensions, to subsidize digital maternal care for refugees and other underserved populations. The proliferation of AI and the increased attractiveness of gamification are elements that support the upscaling of those tools in the future. Having said that, it must be noted that to fully maximize the benefits of such interventions, a holistic approach is needed—one that addresses social determinants of health, standardizes clinical protocols, and ensures continuity of care, while considering context-specific factors. By combining digital innovation with social support, policymakers and healthcare systems can create more equitable and effective maternal healthcare frameworks, ultimately improving long-term outcomes for both mothers and infants.

## Data Availability

The raw data supporting the conclusions of this article will be made available by the authors, without undue reservation.
